# Heart Failure and Frailty in the Community-Living Elderly Population: What the UFO Study Will Tell Us

**DOI:** 10.3389/fphys.2018.00347

**Published:** 2018-04-24

**Authors:** Erik Fung, Elsie Hui, Xiaobo Yang, Leong T. Lui, King F. Cheng, Qi Li, Yiting Fan, Daljit S. Sahota, Bosco H. M. Ma, Jenny S. W. Lee, Alex P. W. Lee, Jean Woo

**Affiliations:** ^1^Department of Medicine and Therapeutics, Faculty of Medicine, Chinese University of Hong Kong, Sha Tin, Hong Kong; ^2^Laboratory for Heart Failure and Circulation Research, Li Ka Shing Institute of Health Sciences, Prince of Wales Hospital, Sha Tin, Hong Kong; ^3^Faculty of Medicine, Gerald Choa Cardiac Research Centre, Chinese University of Hong Kong, Sha Tin, Hong Kong; ^4^Department of Medicine and Geriatrics, Shatin Hospital, Sha Tin, Hong Kong; ^5^PhD Programme in Medical Sciences, Division of Medical Sciences, Department of Medicine and Therapeutics, Faculty of Medicine, The Chinese University of Hong Kong, Shatin, Hong Kong; ^6^Department of Obstetrics and Gynaecology, Faculty of Medicine, Chinese University of Hong Kong, Sha Tin, Hong Kong; ^7^Department of Medicine, Alice Ho Miu Ling Nethersole Hospital and Tai Po Hospital, Tai Po, Hong Kong; ^8^CUHK Jockey Club Institute of Ageing, Chinese University of Hong Kong, Sha Tin, Hong Kong

**Keywords:** heart failure, frailty, elderly, aging, screening

## Abstract

Heart failure and frailty are clinical syndromes that present with overlapping phenotypic characteristics. Importantly, their co-presence is associated with increased mortality and morbidity. While mechanical and electrical device therapies for heart failure are vital for select patients with advanced stage disease, the majority of patients and especially those with undiagnosed heart failure would benefit from early disease detection and prompt initiation of guideline-directed medical therapies. In this article, we review the problematic interactions between heart failure and frailty, introduce a focused cardiac screening program for community-living elderly initiated by a mobile communication device app leading to the Undiagnosed heart Failure in frail Older individuals (UFO) study, and discuss how the knowledge of pre-frailty and frailty status could be exploited for the detection of previously undiagnosed heart failure or advanced cardiac disease. The widespread use of mobile devices coupled with increasing availability of novel, effective medical and minimally invasive therapies have incentivized new approaches to heart failure case finding and disease management.

## Introduction

The word frailty (or fragility) is derived from the Old French word, *fraileté*, or the Latin word, *fragilitas*; and is defined in the Oxford English dictionary as “the condition of being weak and delicate” (Simpson and Weiner, 1989). Frailty is a common geriatric syndrome that predisposes to falls, hospitalization, and death in the elderly (Morley et al., 2013). The salient features of weakness, skeletal muscle wasting (sarcopenia), and exercise intolerance are present in both chronic heart failure (HF) and frailty (Morley et al., 2013; Joyce, 2016). HF is a leading cause for hospital admission, and incurs high recurrent costs to healthcare systems and societies. Because HF with frailty is associated with a worse prognosis including increased rates of hospitalization and death compared with HF without frailty (Cacciatore et al., 2005; Gastelurrutia et al., 2014; Vidán et al., 2016), it is important to identify frail and pre-frail individuals, determine if they have HF and the particular HF subtype (i.e., HF with reduced or preserved ejection fraction), and implement risk modification, guideline-directed therapies and interventions, as appropriate (Writing Committee Members et al., 2013; Ponikowski et al., 2016; Yancy et al., 2017). In patients with chronic HF, multidisciplinary interventions including strength and muscle training, physical rehabilitation, and nutritional supplementation may improve functional impairment and deficits (Piepoli et al., 2004; O'Connor et al., 2009; Davies et al., 2010; Rozentryt et al., 2010; Avni et al., 2012; Gotsman et al., 2012; Taylor et al., 2014). Furthermore, in eligible candidates frailty may be reversible with left ventricular assist device (LVAD) therapy or heart transplant (Jha et al., 2016, 2017; Maurer et al., 2017). However, these advanced therapies are costly and may not be available or suitable for many patients with HF. To effectively manage HF for the majority of patients, a collaborative cardiogeriatric model (Azad and Mielniczuk, 2016) and community-based approach to maximize public awareness (Sacha et al., 2017), case finding, and implementation of guideline-directed therapies is warranted and timely, given the growing HF and aging populations worldwide.

Recently, a cohort study by Lee et al. including 3,018 community-living older adults (aged ≥65 years) identified factors that were associated with adverse transition of frailty status or impediment to improvement, particularly diabetes in women and previous stroke in men (Lee et al., 2014). However, it remains unclear what proportion of community-living older individuals harbored HF and other advanced cardiac disease that develop and progress insidiously. As will be discussed below, the ***U***ndiagnosed heart ***F***ailure in frail ***O***lder individuals (UFO) study aims to uncover the scale of HF in community-living older individuals through focused cardiac screening, and to systematically profile the frailty status and HF phenotype (and subphenotype) of these individuals using formal frailty assessment instruments, physical fitness analysis, and high-dimensional “omics” technologies.

## Epidemiology

The scale of the global aging population is increasingly recognized. Estimates by the United Nations projected that by 2025, there will be 153.4 million individuals aged ≥80 years, and by 2050 there will be 379 million^1^. Equally alarming is the expanding HF population in high-density nations and developing regions of the world including China, India, Southeast Asia, the Middle East and Latin America (Guo et al., 2013; Savarese and Lund, 2017). There aEre at least 26 million individuals with HF worldwide (Ambrosy et al., 2014). In Europe and the United States, the health care expenditure attributed to HF is approximately 1–3% (Cowie et al., 2014; Benjamin et al., 2017; Soundarraj et al., 2017). While cardiovascular mortality from HF has improved in western countries due to disease awareness and implementation of guideline-directed therapies and management (Rush et al., 2015; Gordin and Fonarow, 2016), projections including those by the American Heart Association point to a substantial increase in the HF and aging populations that will lead to direct costs of (at least) US$160 billion for the United States by 2030 (Heidenreich et al., 2013). Using World Bank data from 197 countries covering 98.7% of the world's population, Cook et al. determined that the cost of HF amounted to US$108 billion in 2012 (Cook et al., 2014). Countries that are ill-prepared for the care of aging populations and management of HF are expected to face considerable socioeconomic challenges.

The prevalence of frailty in the general population has been estimated at 4–59%, depending on the study (Collard et al., 2012). The scale of undiagnosed HF in the community-dwelling elderly remains unclear in most populations, as there has not been formal screening designed to specifically assess older individuals with signs and symptoms of frailty for HF. Importantly, frailty has a considerable negative impact on patients with HF, as the presence of frailty increases the risk for both hospitalization and death by at least 1.5-fold (Lupón et al., 2008; Afilalo et al., 2009). In advanced HF patients undergoing LVAD implantation or heart transplantation, preoperative frailty assessment can indicate those with increased postoperative risks of death, prolonged utilization of intensive care unit and length of hospital stay (Jha et al., 2016, 2017). Importantly, frailty can reverse or be improved in some patients with HF (Lee et al., 2014; Jha et al., 2016; Maurer et al., 2017), refuting the notion that frailty is permanent, inevitably irreversible and age-dependent.

The traditional approach to diagnosing and managing HF in the elderly has depended on patient's presentation of symptoms, on incidental findings, or during an index hospitalization for acute decompensated HF. The first hospitalization for HF is considered late in the disease course, as the index event carries a mortality rate of 30–50% in the first year (Blackledge et al., 2003)^2^. The lethality of HF and recent advances in therapeutic options justify proactive, early detection and upstream intervention.

The rationale for screening of suspected frail elderly individuals is supported by available clinical instruments (e.g., Fried frailty assessment, FRAIL scale) (Fried et al., 2001; Abellan van Kan et al., 2008a,b; Morley et al., 2012) and noninvasive technologies (e.g., echocardiography, strain imaging) to detect the syndromes, to provide diagnostic information (e.g., diastolic or systolic dysfunction, advanced valvular heart disease), risk stratification and prognosis (e.g., New York Heart Association functional class, Seattle Heart Failure Model), as well as treatments to modify risks and clinical outcomes (e.g., blood pressure and glucose control, valsartan/sacubitril for HF with reduced left ventricular ejection fraction [HFrEF, LVEF <40%] McMurray et al., 2014, ivabradine for tachycardia in HFrEF Fox et al., 2008, empagliflozin for diabetic patients with HF Zinman et al., 2015; Fitchett et al., 2016), improve symptoms and function (e.g., cardiac resynchronization therapy for eligible patients; Madhavan et al., 2017; Mulpuru et al., 2017) and alter the natural course of the disease (e.g., LVAD as destination therapy; Rose et al., 2001; Slaughter et al., 2009; Mehra et al., 2017). Frail elderly patients with HF remain understudied in clinical trials, and there is often inadequate clinical data to inform evidenced-based practice (Ahmed, 2009). Public health policy for HF and the aging population should therefore refocus on early detection and delivery of prompt therapeutic interventions to forestall the rise in hospitalization, morbidity and deaths.

## Frailty, comorbidities, and undiagnosed heart failure in the community

The frailty concept developed by Rockwood and Mitnitski is based on a multiple deficit approach (Rockwood et al., 2005), utilizing clinical and laboratory characteristics with an emphasis on the number of abnormalities rather than the type, the key requirement being a minimum of 30 variables rather than the type of deficit. Clinical characteristics may include symptoms, functional impairments, and history of different diseases. Frailty is reported as the total number of deficits divided by the number of variables considered and this frailty index (FI) theoretically ranges from 0 to 1 (maximal frailty value). However, there appears to be a ceiling of 0.7 in animal models (0.67 in mice) (Howlett, 2015) and in population studies. Therefore, some symptoms and signs of HF may or may not be covered by the index. It is noteworthy that the FI is mainly used as a research tool, rather than for patient management.

In contrast, frailty as a clinical phenotype, proposed by Fried et al. (2001), is based on a concept of failure of homeostasis with age as a result of abnormalities in physiological systems (Tang et al., 2017), represented by five specific items: low physical activity, fatigue, shrinkage (or weight loss), weakness, and slowness. Each positive answer has a score of 1. A total score of ≥3 represents frailty, while a score of 1–2 represents the pre-frail state. This definition is commonly used but operationalized in different ways in the clinical setting, with or without physical performance measures such as hand grip strength and slow walking speed. One version without the need for measurements was proposed by Malmstrom and Morley (the FRAIL scale) (Morley et al., 2012), which consists of a set of five domain questions: Fatigue (tired all or most of the time), Resistance (difficulty walking up 10 steps without resting or aids), Ambulation (difficulty walking several 100 yards (or approximately 500–600 m) alone without aids), Illness (≥5), Loss of weight (>5% within the past 12 months) (Supplemental Figure 1). One point is assigned for each positive answer. The classification into the three frailty categories is as for the Fried phenotype. The first three questions of the FRAIL scale may be considered symptoms of HF. This approach satisfies the requirement for a multi-domain assessment of frailty in HF, as pointed out by other investigators in the field (Abellan van Kan et al., 2008a,b; McDonagh et al., 2018). In general, both the FRAIL scale and the Fried phenotype reflect failure of homeostasis which may be considered a physiological failure of the heart to adapt to environmental stressors.

The comprehensive geriatric assessment-based Multidimensional Prognostic Index (MPI) reportedly predicts 1-month mortality in older patients with HF (Pilotto et al., 2010). MPI is essentially based on accumulation of the number of deficits, and is similar to the Frailty Index proposed by Rockwood et al., in contrast to the frail phenotype based on physiological dysregulation proposed by Fried. However, in terms of prediction of mortality and physical limitation, a comparison of frailty indicators based on clinical phenotype and the multiple deficit approach showed that the major frailty assessment instruments have similar ability to predict adverse outcomes, with ROC curve values being approximately 0.6 (Woo et al., 2012).

All the above instruments discussed thus far have similar predictive properties in having high specificity but low sensitivity for predicting incident physical limitation and mortality (Woo et al., 2012). Various other instruments consisting of different combinations of physical performance measures, disability indicators, and polypharmacy have been tested and proposed (Hoogendijk et al., 2013; Clegg et al., 2015). However, they do not appear to have clear underlying links with HF.

The prevalence of frailty varies widely, depending on the study population. In 23 studies including 8,871 patients with known chronic HF published in the literature, the mean prevalence of frailty was 44.2% (median 41.7%). In consecutively recruited study subjects, the mean prevalence was 43.5% (median 49.6%) (Table 1). The burden of multiple medical comorbidities is relatively high in chronic HF patients, particularly hypertension (Jin et al., 2014), ischemic heart disease, diabetes and atrial fibrillation (Table 1); other commonly reported comorbidities in HF include anemia, chronic obstructive pulmonary disease and renal insufficiency (Dahlström, 2005; Ather et al., 2012; Murad and Kitzman, 2012; Paulus and Tschöpe, 2013). The increasing number of comorbidities or abnormal organ systems in HF patients positively correlates with both the degree of frailty and increased risks for adverse outcomes including hospitalization and death (Murad and Kitzman, 2012; Nadruz et al., 2017). Treatment of HF and systemic comorbidities have been shown to be associated with improved outcomes (Dahlström, 2005; Avni et al., 2012; Gotsman et al., 2012; Zinman et al., 2015; Fitchett et al., 2016). It remains to be determined whether cardiac screening of frail elderly with multiple comorbidities is cost-effective in discovering previously unrecognized HF or other serious cardiac abnormalities (e.g., severe aortic stenosis amenable to surgical or transcatheter aortic valve replacement, severe mitral insufficiency for valve replacement or repair), and whether that will translate into a reduction in healthcare resource utilization in the long term.

**Table 1:** Characteristics and phenotype of patients from 23 published studies that included chronic heart failure (HF) and frailty.

**Author (publication year)**	**PMID**	**Age, *y* ± SD**	**Prevalence of frailty among HF patients, %**	**Frailty assessment instrument**	**Prevalence of AF, %**	**Prevalence of IHD, %**	**Prevalence of HTN, %**	**Prevalence of DM, %**	**Prevalence of dyslipidemia, %**	**Prevalence of obesity, %**	**BMI (kg/m^2^)^‡^**
Altimir et al., 2005^†^	16087134	65.2 ± 10.9	41.7 (150/360)	CGA	N/A	58.9 (212/360)	N/A	N/A	N/A	N/A	N/A
Boxer et al., 2008	18174754	77 ± 10	25 (15/60)	Fried phenotype	N/A	7(4/60) (angina)	73 (44/60)	32 (19/60)	N/A	N/A	27.8 ± 5.2
Boxer et al., 2010	20887617	78 ± 12	25.4 (15/59)	Fried phenotype	N/A	N/A	N/A	N/A	N/A	N/A	N/A
Cacciatore et al., 2005^*^	16313247	75.9 ± 6.7	15 (18/120)	Frailty staging system	N/A	56.7	N/A	N/A	N/A	N/A	N/A
Ferguson et al., 2017^†^	27036952	72 ± 16	63 (58/92)	SHARE-FI	100 (137/137)	43 (56/129)	64 (85/133)	37 (46/133)	51.9 (68/131)	N/A	N/A
Gastelurrutia et al., 2013^†^	24012028	66.7 ± 12.4	44.2 (621/1,405)	CGA	18.0 (253/1,405)	52.8 (742/1,405)	60.9 (855/1,405)	39.2 (551/1,405)	N/A	N/A	N/A
Gastelurrutia et al., 2014^†^	24820761	66.7 ± 12.4	44.2 (581/1,314)	CGA	18.0 (237/1,314)	53.7 (706/1,314)	60 (789/1,314)	38.4 (505/1,314)	N/A	N/A	N/A
González-Moneo et al., 2016^†^	27577747	71 ± 11	55 (279/509)	Barber questionnaire	N/A	N/A	79 (412/525)	47 (239/525)	N/A	N/A	N/A
Kenny et al., 2006	16770521	Men, 76 ± 9, Women, 78 ± 12	27.6 (16/59)	Fried phenotype	N/A	N/A	N/A	N/A	N/A	N/A	27.7 ± 5.3
Khandelwal et al., 2012^*^	23076517	N/A	76.7 (23/30)	Fried phenotype	N/A	N/A	N/A	N/A	N/A	N/A	N/A
Lupón et al., 2008^†^	18684366	68 (median)	39.9 (248/622)	CGA	N/A	57.9 (360/622)	55.9 (348/622)	39.2 (244/622)	N/A	N/A	N/A
Madan et al., 2016^†^	26883168	74.9 ± 6.5	65 (26/40)	Fried phenotype	42.5 (17/40)	47.5 (19/40)	87.5 (35/40)	55 (22/40)	N/A	N/A	N/A
McNallan et al., 2013b	23956958	73.2 ± 13.3	18.8 (84/448)	Fried phenotype	63.4 (284/448) (AF or flutter)	26.5 (118/448) (prior MI)	90.4 (405/448)	39.2 (175/448)	82.6 (369/448)	N/A	29.7 (26–35)
McNallan et al., 2013a	24093859	71.1 ± 13.9	20.6 (46/223)	Fried phenotype	60.5 (135/223) (AF or flutter)	27 (60/223) (prior MI)	89.7 (200/223)	40.8 (91/223)	81.1 (180/223)	N/A	30.5 (26–37)
Newman et al., 2001^*^	11253157	N/A	23.2 (42/181)	Fried phenotype	N/A	N/A	N/A	N/A	N/A	N/A	N/A
Nishiguchi et al., 2016	27605942	73.7 ± 7.3	16.5 (34/206)	Fried phenotype	N/A (arrhythmia, 21.8, 45/206)	89.3 (184/206) (angina and MI)	48.5 (100/206)	20.4 (42/206)	34.5 (71/206)	N/A	23.5 ± 3.3
Pons et al., 2010^†^	20196991	69	35.1 (337/960)	CGA	16.9 (162/960)	55.4 (532/960)	58.6 (563/960)	39.3 (377/960)	42.8 (411/960)	N/A	27.1 (24.1–30.5)
Rodríguez-Pascual et al., 2017	28215465	85.2 ± 7.3	57.5 (286/497)	Fried phenotype	61.0 (303/497)	23.5 (117/497)	82.3 (409/497)	32 (159/497)	N/A	N/A	N/A
Sánchez et al., 2011	21795299	81.6 ± 5	40.8 (86/211)	Fried phenotype	39.3 (83/211)	45 (95/211) (MI)	80.1 (169/211)	42.2 (89/211)	43.1 (91/211)	N/A	N/A
Uchmanowicz and Gobbens, 2015	26491276	Non-frail, 62.3 ± 6.2, Frail, 67.9 ± 10.7	89 (89/100)	TFI	N/A	N/A	N/A	N/A	N/A	N/A	N/A
Vidán et al., 2014^†^	25516357	80.1 ± 6.1	70.2 (316/450)	Fried phenotype	53.3 (240/450)	34.4 (155/450)	87.7 (392/450)	34 (152/450)	53.6 (238/450)	N/A	N/A
Vidán et al., 2016^†^	27072307	80 ± 6.1	76 (316/416)	Fried phenotype	52.4 (218/416)	32.7 (136/416)	87.0 (362/416)	33.7 (140/416)	N/A	N/A	Frailty: 27.1 ± 5.6, Non-frailty: 26.9 ± 5.1
Woods et al., 2005^*^	16078957	N/A	45.6 (232/509)	Fried phenotype	N/A	N/A	N/A	N/A	N/A	N/A	N/A
Mean	–	–	44.2 (3,918/8,871)	N/A	33.5 (2,114/6,307)	47.6 (3,496/7,341)	68.8 (5,168/7,510)	38 (2,851/7,510)	54.3 (1,428/2,629)	N/A	N/A

*AF, atrial fibrillation; BMI, body mass index; CGA, Comprehensive Geriatric Assessment (including Barthel Index, Older Americans Resources and Services Scale, Pfeiffer Test, and the abbreviated Geriatric Depression Scale); DM, diabetes mellitus; HTN, hypertension; IHD, ischemic heart disease; MI, myocardial infarction; N/A, not applicable; PMID, PubMed unique identifier; SHARE-FI, The Survey of Health, Ageing and Retirement in Europe (SHARE) Frailty Index (FI); TFI, Tilburg Frailty Indicator*.

* *Only patients with HF analyzed*.

† *Consecutively recruited patients with chronic HF*.

‡ *BMI is shown as mean ± standard deviation, or median (interquartile range)*.

To date, two cross-sectional studies from the Netherlands have provided some clues on the scale of undiagnosed HF in patients with suggestive symptoms. Although those studies did not include frailty assessment and recruited subjects based on symptoms (e.g., exertional dyspnea), HF was diagnosed by cardiologists and verified by echocardiography and other investigations. Oudejans et al. evaluated 206 elderly patients (mean age 82 years) referred to geriatric outpatient clinics for assessment of HF symptoms and diagnosed HF in 46% of study subjects (Oudejans et al., 2011). In another study on 585 older individuals (age ≥65 years) presenting to 30 general practices with chronic exertional dyspnea and no prior history of HF, van Riet et al. diagnosed HF in 15.7% of the subjects (van Riet et al., 2014). Those findings suggest that HF may be present in one-eighth to half of the elderly in the community experiencing “typical” HF symptoms, which are often difficult to distinguish from those of frailty.

## Undiagnosed heart failure in frail older individuals: the UFO study

Recently, our group has launched the UFO study to gain an understanding of the extent of frail older individuals with previously unknown HF. The UFO study was developed following a territory-wide wellbeing survey (WBS) project^3^ using the FRAIL scale (Morley et al., 2012; Woo et al., 2012; Supplemental Figure 1) presented in the form of a mobile device app, which detected 18.63% frail older individuals aged 60 years and over among 2,539 respondents, and 45.96% pre-frail individuals; among the local hospital geriatric population the prevalence of frailty has been estimated at approximately 50% (unpublished data). Work is underway to compile mobile device app data on 4,000+ individuals. Interim data on the characteristics and demographics of the questionnaire respondents are shown in Table 2. As HF will be verified using clinical and functional assessments, echocardiography and N-terminal pro B-type natriuretic peptide (NT-proBNP) levels, the number of unrecognized HF patients residing in the community may be substantial.

**Table 2:** Interim analysis of characteristics and demographics of Hong Kong respondents (*n* = 2,539) in the Wellbeing Survey via mobile device app.

**Characteristics**	**Subgroups**	**Number (percentage of total)**
Age distribution, *n*	<60 years	7 (0.28%)
	60–69 years	608 (23.95%)
	70–79 years	1,088 (42.85%)
	≥80 years	836 (32.93%)
Sex	Male	603 (23.7%)
	Female	1,936 (76.3%)
Frailty status^*^	Robust (0 points)	899 (35.4%)
	Pre-frail (1–2 points)	1,167 (46%)
	Frail (3–5 points)	473 (18.6%)
Body mass index^†^, kg/m^2^	<18.5	34 (3.5%)
	18.5–22.9	281 (28.5%)
	23–26.9	406 (41.2%)
	≥27	264 (26.8%)
Self-reported comorbidities, *n*	Hypertension	1,683 (66.29%)
	Diabetes mellitus	714 (28.12%)
	Dyslipidemia	705 (22.77%)
	Coronary artery disease	399 (15.71%)
	Stroke	112 (4.41%)
	Chronic obstructive lung disease	82 (3.23%)
	Kidney disease	40 (1.58%)
Ideal (normal) blood pressure, SBP 90–120/DBP 60–80 mmHg	Yes	131 (11.9%)
	No	974 (88.1%)

*SBP, systolic blood pressure. DBP, diastolic blood pressure*.

* *Assessed by self-administered FRAIL scale (out of 5 points) on mobile device app*.

*For details of the FRAIL scale, please refer to the text and Supplemental Figure 1 (Abellan van Kan et al., 2008a,b; Morley et al., 2012)*.

† *Body mass index cut-points for Asians (Lancet 2004; 363:157–163)*.

It is noteworthy that HF in older individuals may present with atypical symptoms other than exertional dyspnea, reduced exercise tolerance or lack of energy. The questions embedded in the FRAIL (Fatigue, Resistance, Ambulation, Illnesses, and Loss of weight) scale (Morley et al., 2012; Supplemental Figure 1) can additionally enhance the clinician's ability to uncover frailty through documentation of multiple medical comorbidities (≥5 illnesses out of 11 total illnesses) and weight decline (unintentional reduction by ≥5%). The FRAIL scale is easy to use, translating into improved operationalization, and has been demonstrated to have similar performance compared with other major frailty assessment instruments in diagnosing frailty, predicting death and/or gauging physical limitation (Woo et al., 2012; Chong et al., 2017).

The UFO study is currently recruiting mobile device app-screened subjects, other elderly in the community, and geriatric patients with frailty following discharge from hospitals within a regional hospital cluster network (New Territories East Cluster of the Hospital Authority, Hong Kong SAR). Frail geriatric patients discharged from hospitals may serve as a benchmark for the high-risk frail group. The study attempts to encompass and recruit as diverse a community-dwelling elderly population as possible, with different health and socioeconomic status in order to minimize bias. However, real-world recruitment bias may be impossible to eliminate; for instance, the frail elderly living and struggling in the community may be less likely or interested to come forth for the study recruitment. Nevertheless, the pre-frail older individuals may be an especially important group to study, given the increasingly available interventional options and the possibility of a successful intervention on preventing progression into the frail state. In this study, apart from clinical assessment, echocardiography and NT-proBNP levels, we will also measure physical fitness (e.g., 6-min walk test, hand grip strength), and compare patients' medication inventory, cardiovascular history and risk profiles, and social determinants of health among different frail/non-frail states.

Recently, investigators of the biracial (white and African American) Atherosclerosis Risk in Communities (ARIC) study have reported an association between frailty and cardiac abnormalities (e.g., left ventricular hypertrophy, reduced global longitudinal strain, increased left atrial volume index) in older adults (Norby et al., 2016). The authors pointed out that measurable dysfunction in the cardiac system stood out most compared with other organ systems or tissues (vascular, pulmonary, renal, hematologic and adipose tissue) investigated. Interestingly, the prevalence of frailty in the ARIC cohort, a study population not particularly known to have low lifetime cardiovascular risks (Norby et al., 2016), was calculated to be between 5.29% (among 3,991 participants, mean age 75.6 ± 5.6 years) (Nadruz et al., 2017) and 6.5% (among 6,080 participants, median age 75 years) (Kucharska-Newton et al., 2017), representing a three-fold lower prevalence compared with our WBS screening data (18.63%, *n* = 2,539 Hong Kong Chinese respondents, median age band of 70–79 years). Aside from minor differences in the frailty assessment instrument used (Fried frailty assessment in ARIC vs. FRAIL scale in UFO; both used an ordinal scale out of 5 points with similar assessment components), it is unclear whether social, cultural and/or ethnic factors might be explanatory.

Of particular interest in the UFO study are frail and pre-frail participants who might harbor HF or other advanced cardiac pathologies with no overt or subclinical symptoms. It is recognized that patients with HF develop ways to adapt to and cope with physical or functional impairment, and change their behavior and perception to compensate for deficits (Yu et al., 2008; Chaves and Park, 2016). It is envisaged that findings from the UFO study will guide us to objectively focus on a subset of elderly in the community requiring medical attention, appropriate referral and intervention if necessary.

## Conclusion

HF and frailty are overlapping clinical syndromes that are prevalent in the elderly. The scale of undiagnosed HF in frail and pre-frail older individuals in the general population remains unclear. UFO is a community-based cardiogeriatric research study designed to uncover the extent of the problem, and will report the detection yield of cardiac screening for HF and serious, potentially actionable cardiac abnormalities in the elderly with or without frailty. Findings from UFO may support early detection and treatment of HF and advanced cardiac disease sequestered in the community.

## Ethics statement

The described study is being carried out under the oversight of the Joint Chinese University of Hong Kong-New Territories East Cluster Clinical Research Ethics Committee (CUHK-NTEC CREC, http://www.crec.cuhk.edu.hk/) with written informed consent from all subjects. All subjects gave written informed consent in accordance with the Declaration of Helsinki. The protocol was approved by the CUHK-NTEC CREC.

## Author contributions

EF: conceived the framework and contents of the paper, collected the data and performed data analysis, wrote the draft, performed editing and is the corresponding author; EH: critically reviewed the manuscript and contributed to the editing of the manuscript; XY: performed data analysis, and contributed to editing; LL, KC, QL, YF, DS, BM, JL, and AL: critically reviewed the manuscript and contributed to editing; JW: contributed to drafting of the manuscript, critically reviewed it and contributed to editing.

### Conflict of interest statement

The authors declare that the research was conducted in the absence of any commercial or financial relationships that could be construed as a potential conflict of interest.

## Footnotes

^1^Population Division DESA and United Nations. “Demographic profile of the older population,” in *World Population Ageing 1950-2050*, 23–27. Available online at: http://www.un.org/esa/population/publications/worldageing19502050/pdf/90chapteriv.pdf (Accessed 26 December, 2017).

^2^Health and Social Care Information Centre. CCG Outcomes Indicator Set. England, June 2015. Available online at: http://digital.nhs.uk/catalogue/PUB17777 (Accessed 30 December, 2017).

^3^Hong Kong Jockey Club (2016). *Club-Initiated e-Health Project to Help Promote Self-Management of the Elderly*. Available online at: http://www.hkjc.com/english/corporate/racing_news_item.asp?in_file=/english/news/2016-11/news_2016112401520.html (Accessed 30 December, 2017).
