# Supplementary material for: Heart Failure and Frailty in the Community-Living Elderly Population: What the UFO Study Will Tell Us
Source: Front Physiol. 2018 Apr 24;9:347. doi: 10.3389/fphys.2018.00347 (PMC5928128; doi:10.3389/fphys.2018.00347)
Supplement: Supplementary file 1 [file Data_Sheet_1.pdf]

Supplemental Figure 1. The FRAIL scale.

I. Components and definitions:

F - **Fatigue**. “How much of the time during the past 4 weeks did you feel tired?”
1 = All of the time, 2 = Most of the time, 3 = Some of the time, 4 = A little of the time, 5 = None of the time.
Responses of “1” or “2” are scored as 1 and all others as 0.

R - **Resistance**. “By yourself and not using aids, do you have any difficulty walking up 10 steps without resting?”
1 = Yes, 0 = No.

A - **Ambulation**. By yourself and not using aids, do you have any difficulty walking several hundred yards?”
1 = Yes, 0 = No.

I - **Illnesses**. For 11 illnesses, participants are asked, “Did a doctor ever tell you that you have [illness]?”
1 = Yes, 0 = No.
The total illnesses (0–11) are recoded as 0–4 = 0 and 5–11 = 1.
The illnesses include hypertension, diabetes, cancer (other than a minor skin cancer), chronic lung disease, heart attack, congestive heart failure, angina, asthma, arthritis, stroke, and kidney disease.

L - **Loss of weight**. “How much do you weigh with your clothes on but without shoes? [current weight]”
“One year ago in (MO, YR), how much did you weigh without your shoes and with your clothes on? [weight 1 year ago]”
Percent weight change is computed as: [(weight 1 year ago – current weight) ÷ weight 1 year ago]] × 100.
Percent change >5 (representing a 5% loss of weight) is scored as 1 and <5 as 0.

II. Score range: 0 to 5 (0 = best to 5 = worst)

III. Classification: Frail (3 to 5), pre-frail (1 to 2), robust (0)

Source: Morley JE, Malmstrom TK, Miller DK. *J Nutr Health Aging* 2012;16:601-608.
